# Epigenetic and post‐transcriptional regulation of somatostatin receptor subtype 5 (SST_5_) in pituitary and pancreatic neuroendocrine tumors

**DOI:** 10.1002/1878-0261.13107

**Published:** 2021-10-26

**Authors:** Sergio Pedraza‐Arevalo, Alejandro Ibáñez‐Costa, Ricardo Blázquez‐Encinas, Miguel R. Branco, Mari C. Vázquez‐Borrego, Aura D. Herrera‐Martínez, Eva Venegas‐Moreno, Raquel Serrano‐Blanch, Álvaro Arjona‐Sánchez, María A. Gálvez‐Moreno, Marta Korbonits, Alfonso Soto‐Moreno, Manuel D. Gahete, Marika Charalambous, Raúl M. Luque, Justo P. Castaño

**Affiliations:** ^1^ Maimonides Institute for Biomedical Research of Cordoba (IMIBIC) Córdoba Spain; ^2^ Department of Cell Biology, Physiology, and Immunology University of Córdoba Córdoba Spain; ^3^ CIBER Fisiopatología de la Obesidad y Nutrición (CIBERobn) Córdoba Spain; ^4^ Reina Sofia University Hospital Córdoba Spain; ^5^ Blizard Institute Barts and the London School of Medicine and Dentistry Queen Mary University of London London UK; ^6^ Endocrinology and Nutrition Service Reina Sofia University Hospital Córdoba Spain; ^7^ Metabolism and Nutrition Unit Hospital Universitario Virgen del Rocío Instituto de Biomedicina de Sevilla (IBIS) Sevilla Spain; ^8^ Medical Oncology Service Reina Sofia University Hospital Córdoba Spain; ^9^ Surgery Service Reina Sofia University Hospital Córdoba Spain; ^10^ Centre for Endocrinology William Harvey Research Institute Barts and the London School of Medicine and Dentistry Queen Mary University of London London UK; ^11^ Developmental Epigenetics group Department of Medical and Molecular Genetics King’s College of London London UK

**Keywords:** epigenetics, natural antisense transcript, neuroendocrine tumors, pancreas, pituitary, SST5

## Abstract

Somatostatin receptor subtype 5 (SST_5_) is an emerging biomarker and actionable target in pituitary (PitNETs) and pancreatic (PanNETs) neuroendocrine tumors. Transcriptional and epigenetic regulation of *SSTR5* gene expression and mRNA biogenesis is poorly understood. Recently, an overlapping natural antisense transcript, *SSTR5‐AS1*, potentially regulating *SSTR5* expression, was identified. We aimed to elucidate whether epigenetic processes contribute to the regulation of *SSTR5* expression in PitNETs (somatotropinomas) and PanNETs. We analyzed the *SSTR5*/*SSTR5‐AS1* human locus *in silico* to identify CpG islands. *SSTR5* and *SSTR5‐AS1* expression was assessed by quantitative real‐time PCR (qPCR) in 27 somatotropinomas, 11 normal pituitaries (NPs), and 15 PanNETs/paired adjacent (control) samples. We evaluated methylation grade in four CpG islands in the *SSTR5*/*SSTR5‐AS1* genes. Results revealed that *SSTR5* and *SSTR5‐AS1* were directly correlated in NP, somatotropinoma, and PanNET samples. Interestingly, selected CpG islands were differentially methylated in somatotropinomas compared with NPs. In PanNETs cell lines, *SSTR5‐AS1* silencing downregulated *SSTR5* expression, altered aggressiveness features, and influenced pasireotide response. These results provide evidence that *SSTR5* expression in PitNETs and PanNETs can be epigenetically regulated by the *SSTR5‐AS1* antisense transcript and, indirectly, by DNA methylation, which may thereby impact tumor behavior and treatment response.

## Graphical abstract

Somatostatin receptor subtype 5 (SST_5_) is a key player in neuroendocrine tumors (NETs). Yet, its regulation is still insufficiently understood. This study presents original evidence that *SSTR5* expression may be epigenetically regulated by the *SSTR5‐AS1* antisense transcript and DNA methylation in pituitary and pancreatic NETs, providing a new regulatory layer influencing receptor levels and, thereby, tumor behavior and treatment response.

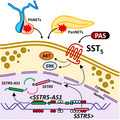

**Abbreviations**
FBS: fetal bovine serum
NAT: natural antisense transcript
NET: neuroendocrine tumor
NP: normal pituitary
NTAT: nontumor adjacent tissue
PanNET: pancreatic NET
PitNET: pituitary NET
SSA: somatostatin analogue

## 1 Introduction

Neuroendocrine tumors (NETs) comprise a heterogeneous group of neoplasms, with rising incidence over the last decades [1, 2, 3]. These tumors arise from cells of (neuro)endocrine origin, which share common features like the synthesis, storage, and secretion of hormones and neurotransmitters. NETs can be widely distributed throughout the body, although they are more abundant in the gastrointestinal and respiratory tracts [1, 2, 3]. Specifically, pancreatic NETs (PanNETs), which display one of the highest increases in incidence within the different types of NETs in the last 10 years [4], are associated with the endocrine compartment of the pancreas. In fact, PanNETs have been classically thought to be derived from hormone‐producing cells of the pancreatic Langerhans’ islets [5], although recent evidence has arisen challenging this concept, and it is presently under debate whether NETs can in fact be originated from a common cell progenitor from the pancreas [6]. Genetic alterations contributing to PanNETs tumorigenesis include frequent mutations in *MEN1*, *ATRX,* or *DAXX* genes [7].

Additionally, tumors derived from the anterior pituitary have been classically termed adenomas due to their nonmetastatic behavior [8]. However, based on their potential aggressiveness and associated morbimortality, the International Pituitary Pathology Club recently proposed to reclassify this pathology and to name them as pituitary neuroendocrine tumors or PitNETs [9], although some controversies have arisen for this nomenclature [10, 11]. Autopsy and imaging studies reveal that PitNETs are the most common intracranial neoplasms (prevalence 10–22%) [12]. PitNETs are primarily classified according to their size and accompanying hormonal hypersecretion [8]. Among them, somatotropinomas arise from somatotropes and oversecrete growth hormone (GH), causing gigantism (in children/adolescents) or acromegaly, characterized by extremity enlargement, facial and skeletal changes, and metabolic, gastrointestinal, cardiovascular, and respiratory complications [13, 14].

A common feature shared by most NETs is the key role played by somatostatin and its receptors (SST_1_‐SST_5_) in their pathophysiological regulation and medical treatment, which is particularly relevant in PitNETs and PanNETs [13, 14, 15, 16, 17]. Indeed, somatostatin represents the main inhibitory signal for normal somatotropes and endocrine pancreatic cells, where it decreases hormone secretion [16, 18, 19]. Importantly, somatostatin also acts on tumor cells inhibiting hormone hypersecretion and cell proliferation, as reported in different tumor types including somatotropinomas, PanNETs, and thyrotropinomas, which abundantly express SSTs [13, 14, 15, 16, 17]. In general, SST_2_ is the most expressed receptor in tumors, followed by SST_5_, with high tumor specificity [20]. Of note, the truncated SST_5_ splicing variant, SST_5_TMD4, has also been found to be notably expressed in several endocrine‐related tumors, particularly PitNETs and PanNETs [21, 22]. Therein, SST_5_TMD4 has been associated with tumorigenesis and malignancy features, likely by playing an inhibitory role over SST_2_ and canonical, full‐length SST_5_ [23, 24, 25]. Elucidating the regulation and interplay of SST_2_ and SST_5_ is particularly important given their key role in the NETs response to treatment with synthetic somatostatin analogues (SSAs) such as octreotide, lanreotide, or pasireotide [2].

First‐generation SSAs (octreotide and lanreotide) preferentially target SST_2_, with less affinity to SST_5_—and, octreotide, SST_3_—and negligible binding for the other SSTs. These drugs have been widely used in the treatment of GH‐ and TSH‐secreting PitNETs and also in PanNETs, to reduce hormonal secretion, control tumor volume, and improve patient symptoms [26, 27, 28]. Nonetheless, a substantial proportion of patients are or become resistant to these treatments [29, 30]. Consequently, a second generation of SSAs with multireceptor binding affinity was developed, based on the idea that simultaneous targeting of several SST, like natural somatostatin, could improve effectiveness in unresponsive patients. From this group, the most widely used compound is pasireotide, showing high affinity to SST_5_, SST_2_, SST_3,_ and SST_1_ [31, 32]. However, SSAs actions do not only depend on their differential binding to specific SSTs. Actually, in somatotropinomas, although the complete set of factors defining SSA responsiveness is not yet fully defined, various specific tumor features and molecular markers have been shown to relevantly influence tumor response to SSAs, including granulation pattern, *AIP* and *GNAS* mutations, β‐arrestin, filamin A, and E‐cadherin expression, as well as, interestingly, *SSTR2*/*SSTR5* expression balance and SST_5_TMD4 presence [13, 16, 33, 34]. Thus, it is important to understand the mechanisms governing the expression of the *SSTR5* gene and its resultant receptor variants (SST_5_, SST_5_TMD4, SST_5_TMD5), for they may impact NETs response to SSAs.

Gene expression is known to be regulated by multiple factors, among which extrinsic factors, such as epigenetic mechanisms, have gained great attention in recent years. A prime epigenetic modification is DNA methylation, which is based on the addition of a methyl group to a cytosine preceding a guanine (CpG). CpG residues are enriched at CpG islands, regions of the genome frequently associated with promoter function. Likewise, noncoding RNAs may act as modular epigenetic regulators [35]. A particular type of noncoding RNAs comprise natural antisense transcripts (NATs) [36], that is, transcripts derived from the opposite strand to a protein‐coding or sense gene, which can regulate the transcription of their corresponding sense genes. NATs importance is rising as sequencing technologies improve, and recent studies are deciphering NATs role in different diseases, including PitNETs [37], where they play distinct roles, like *AFAP1‐AS1*, which influences tumor growth, or *C5orf66‐AS1*, related to invasiveness. Recently, a NAT for *SSTR5* was reported to be expressed in laryngeal squamous cell carcinoma, where it may act as tumor suppressor [38]. Nevertheless, its role in PitNETs and PanNETs has not been explored yet.

Consequently, in this study we aimed to widen our still limited knowledge of the epigenetic mechanisms underlying the regulation of *SSTR5* expression in NETs, specifically somatotropinomas and PanNETs, and to explore the functional and pathological implications of those epigenetic underpinnings in tumor behavior to better understand the role of this receptor.

## 2 Material and methods

### 2.1 Patients and samples

This study was carried out within a project approved by the Research Ethics Committee of Coórdoba (*Comité de Ética de la Investigación de Córdoba*) and was conducted in accordance with ethical standards of the Helsinki Declaration of the World Medical Association. Written informed consent was obtained from each patient. Pituitary samples were collected during transsphenoidal surgery from 27 acromegaly patients and 11 normal pituitaries (NPs) by autopsy from donors and were stored frozen. Formalin‐fixed paraffin‐embedded samples (FFPE, *n* = 15) were obtained from primary PanNETs; nontumor adjacent tissue, used as control, was extracted from the same piece and both tissues were separated by expert pathologists (patient features summarized in Table S1).

### 2.2 Cell culture and treatment

Functional assays were performed in PanNET model cell lines BON‐1 and QGP‐1 [39, 40, 41, 42], using passages lower than 25 in all cases. BON‐1 cells were kindly provided by Dr. M.C. Zatelli and were cultured in DMEM‐F12 (Life Technologies, Barcelona, Spain), whereas QGP‐1 cells were kindly provided by Dr. K. Öberg and were cultured in RPMI‐1640 (Life Technologies), both supplemented with 10% fetal bovine serum (FBS; Sigma‐Aldrich, Madrid, Spain) and 0.2% antibiotic (Gentamicin/Amphotericin B; Life Technologies). Cell lines were grown at 37 °C, in a humidified atmosphere with 5.0% CO_2_ and were verified for mycoplasma contamination by PCR with specific mycoplasma primers. To ensure the identity of the cells, we could not employ typical STR tests, as they are not available for these cell lines. Therefore, we use a different strategy, by measuring an ample set of genes typically expressed by the cell lines as previously reported [39, 40], including SSTs, and secretory products (e.g., chromogranin, serotonin, or somatostatin). In addition, we have tested cell responses and behaviors after classic treatments, which closely resembled those described by original studies [41, 42]. Pasireotide was provided by Novartis and administered at 100 nm, dissolved in sterile water, as previously reported [31, 43], and 5‐azacytidine (Sigma‐Aldrich) was administered at different doses, based on the literature [44], also dissolved in sterile water.

### 2.3 Silencing of *SSTR5‐AS1* and *SSTR5* expression

BON‐1 and QGP‐1 cells were transfected with a specific shRNA targeting *SSTR5‐AS1*, previously validated in our laboratory (Origene, Rockville, MD, USA), and selected with puromycin. On the other hand, *SSTR5* was transiently silenced with a specific siRNA (Thermo Fisher, Waltham, MA, USA). Specifically, cells were seeded in 6‐well culture plates and transfected with 1 μg of the small RNA, using Lipofectamine 2000 and Lipofectamine RNAiMAX Transfection Reagents (Thermo Fisher) for the shRNA and siRNA, respectively, during 6 h. Scramble shRNA/siRNA served as control.

### 2.4 DNA and RNA isolation and retrotranscription

Total RNA from cell lines was isolated using TRIzol Reagent (Sigma‐Aldrich) treated with DNase (Promega, Barcelona, Spain). In FFPE samples, RNA was isolated RNeasy FFPE Kit (Qiagen, Limburg, Netherlands). Particularly genomic DNA and RNA from fresh pituitary samples were extracted using AllPrep DNA/RNA/Protein Kit (Qiagen). Nucleic acid amount and quality was determined using NanoDrop2000 spectrophotometer (Thermo Fisher) and reversely transcribed using random hexamer primers with the First Strand Synthesis Kit (Thermo Fisher).

### 2.5 Quantitative real‐time PCR (qPCR)

qPCRs were performed using Mx3000p system with the Brilliant III SYBR Green Master Mix (Stratagene, La Jolla, CA, USA) with specific primers (Table S2a) [45]. Results were validated as previously reported [46], adjusting gene expression with a normalization factor, calculated from values of *ACTB*, *GAPDH*, *HPRT1,* and/or *RNA18S1* control genes.

### 2.6 Methylation assay

DNA methylation of CpG islands overlapping *SSTR5* and *SSTR5‐AS1* was evaluated in the PitNETs and normal pituitary cohort, as well as BON‐1 and QGP‐1 cell lines. One µg genomic DNA was used following a protocol previously reported [47] using EZ DNA methylation‐Gold kit (Zymo, Irving, CA, USA). Primers were designed using pyromark software (Qiagen; Table S2b) for 300 bp amplicons, approximately. These primers included Illumina sequencing adaptors, used for a second‐round PCR, which was then performed to index each pituitary sample. Samples were pooled, purified, and size selected with AmpPure beads (Beckman‐Coulter, Brea, CA, USA) and sequenced using the Illumina MiSeq v2 300 cycle run kit. Paired‐end reads were mapped using Bismark to a custom genome made up of the amplicon sequences. An R script was then used to extract average methylation values for each CpG position. Methylation levels from multiple CpGs were then averaged to produce a value per amplicon, excluding positions where mutations/deletions at CpGs were frequently observed in patients; specifically, the first five CpGs were used for CpG1 and CpG2; the first eight CpGs in CpG4.1; and all CpGs in the remaining regions.

### 2.7 Proliferation, colony formation, and migration

Proliferation, colony formation, and cell migration assays were performed as previously described [45, 48]. Briefly, BON‐1 proliferation and colony formation were performed by seeding 1000 cells in 6‐well plates for 10 days. For proliferation, cells were treated 24 h after seeding and refreshed every 48 h; for colony formation, treatment was made only during 24 h prior to seeding. QGP‐1 proliferation assay was performed using Alamar Blue Reagent (Bio‐Source International, Camarillo, CA, USA), as previously reported [45]. Cell migration was evaluated by wound‐healing assay, seeding cells in 24‐well plates until maximum confluence. Then, we made a scratch in the middle on the well and took images of the scratch at 0 and 24 h. Wound healing was calculated as the uncovered area 24 h after the wound compared to the uncovered area just after wounding. Wound‐healing assay is not feasible in QGP‐1 cell line since these cells grow in clusters and do not migrate to fill out the empty space made on the plate surface.

### 2.8 Western Blot

BON‐1 and QGP‐1 cells transfected and treated were lysed to analyze protein phosphorylation by western blot, following standard procedures [49], and using phospho‐ERK (#4370S, Cell Signaling, Beverly, MA, USA), phospho‐AKT (#4060S, Cell Signaling), AKT (#9272S, Cell Signaling), and ERK (sc‐154, Santa Cruz Biotechnology, Dallas, TX, USA) antibodies and HRP‐conjugated goat‐anti rabbit (#7074s; Cell Signaling) secondary antibody. Primary antibodies were diluted 1 : 1000, and secondary antibody was used at 1 : 2000. Band densitometry analysis was performed with imagej software (Bethesda, MD, USA), using total protein as reference factor of corresponding phosphorylated protein.

### 2.9 Statistical analyses

Statistical comparisons between groups were performed by unpaired parametric *t* test and nonparametric Mann‐Whitney *U* test, according to normality (Kolmogorov‐Smirnov test). Pearson’s or Spearman’s bivariate correlations were performed for quantitative variables. One‐way ANOVA analysis was used for the statistical comparison between more than two groups, since all of them were normally distributed (Kolmogorov–Smirnov or Shapiro–Wilk tests). The *P*‐values were two‐sided, and statistical significance was considered when *P* < 0.05. Statistical analyses were assessed using graphpad prism 7 (GraphPad Software, La Jolla, CA, USA).

## 3 Results

### 3.1 Role of DNA methylation and natural antisense transcript (NAT) in the regulation of *SSTR5* transcription in somatotropinomas and PanNETs

**Fig. 1:**
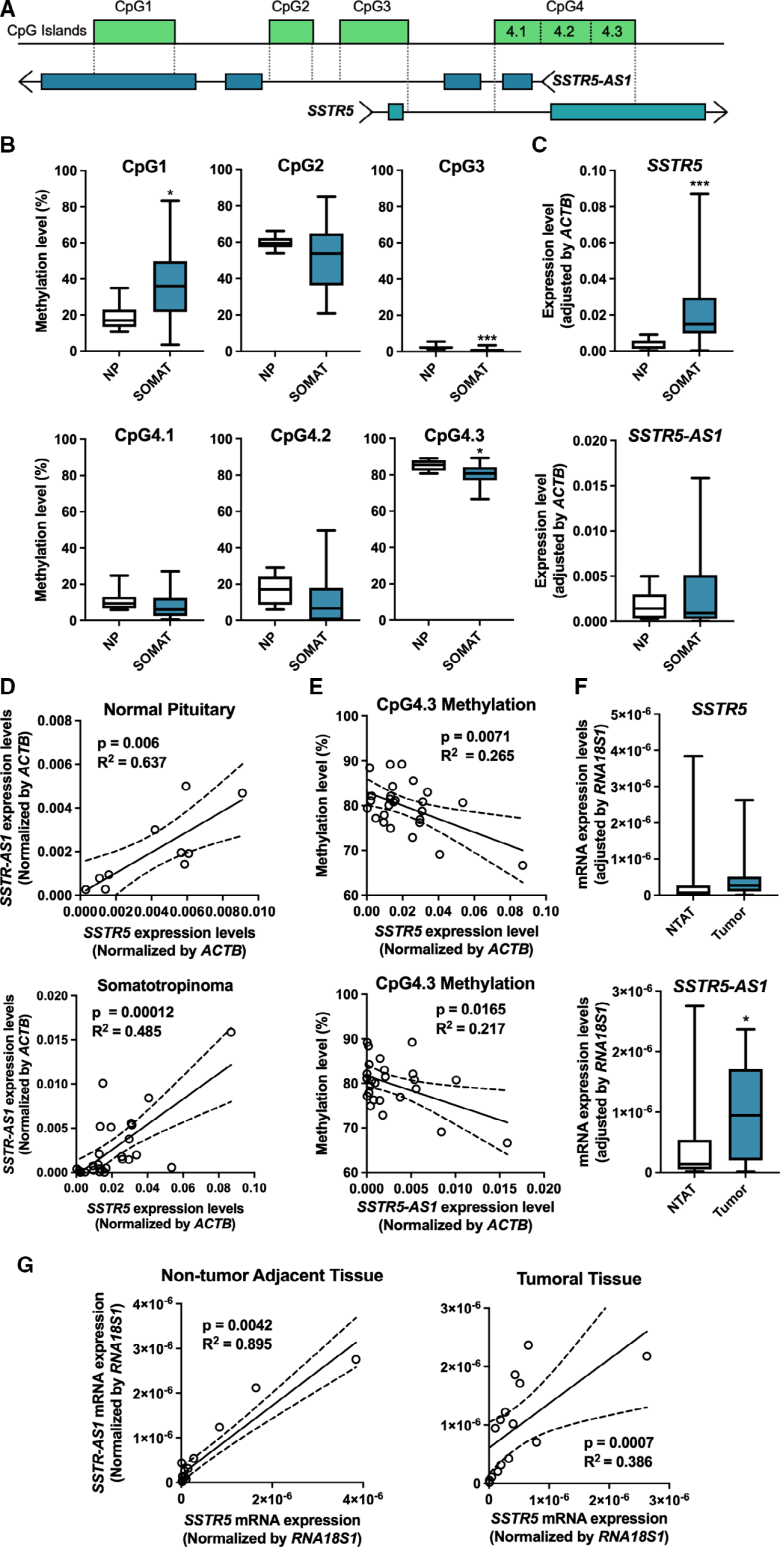
Expression of *SSTR5* is regulated by DNA methylation and NAT. (A) Cartoon representation of *SSTR5‐AS1* and *SSTR5* loci, based on Genome Browser information. (B) Comparison of methylation levels between somatotropinoma (SOMAT) and normal pituitary (NP) samples, expressed as percentage, under *t* test. (C) Expression levels of *SSTR5* and *SSTR5‐AS1* (*t* test) and (D) correlations (Pearson correlation) between them in somatotropinomas and NPs, measured by qPCR and normalized by *ACTB*. (E) Correlations (Pearson correlation) between methylation levels of CpG4.3 and expression levels of *SSTR5* and *SSTR5‐AS1* in somatotropinoma samples. (F) Expression levels of *SSTR5* and *SSTR5‐AS1* (Mann–Whitney *U* test) and (G) correlations (Spearman correlation) between them in PanNETs and nontumor adjacent tissue (NTAT), measured by qPCR and normalized by *RNA18S1*. Asterisks (*P < 0.05; ***P < 0.001) indicate values that significantly differ from control. In all cases, data represent median and interquartile range of 27 somatotropinomas, 11 NPs and 15 PanNETs with their NTAT.

As an initial approach, we performed an *in silico* study of the structure of the *SSTR5* gene (Fig. 1A, Fig. S1). The information obtained from the UCSC Genome Browser (version GRCh37/hg19) revealed the existence of an overlapping gene in humans, *SSTR5‐AS1*, which encodes a long intergenic noncoding RNA, and could regulate *SSTR5* expression, as has been shown for other NATs. Moreover, there are four CpG islands, named hereafter as CpG1‐4, which are susceptible zones of methylation, along both genes, which could also regulate their expression. Some of those CpG islands are in sites of interest, for they could be important in the control of the expression of these genes. Specifically, CpG1 overlaps with the last exon of the NAT and CpG2 falls on the big intron of NAT. CpG3 coincides with the first exon of the *SSTR5* gene, partially overlapping with its promoter, and with another part of the larger intron of the *SSTR5‐AS1*. Besides, CpG4 was the largest region identified and was subdivided into three subzones for the purpose of the study: CpG4.1 overlaps with the start of the NAT, possibly with its promoter, and the intron of *SSTR5*; CpG4.2 falls in the exon of *SSTR5* and coincides with the coding sequence of the canonical SST_5_; CpG4.3 overlaps with the center of the large exon of *SSTR5* gene, including its zone of alternative splicing, and the zone immediately previous to the *SSTR5‐AS1* gene.

In the first experimental assay, we used bisulfite sequencing to measure the methylation levels of these four CpG zones (Fig. 1B) in a cohort of 11 normal pituitary (NP) samples and 27 samples of somatotrope tumors causing acromegaly (summarized in Table S1). Specifically, CpG1 was 20% more intensely methylated in somatotropinomas than in NP. In contrast, CpG3, which displayed levels of < 5% of methylation in all the samples, exhibited a marginally lower, but significant, degree of methylation in somatotropinoma than in NP samples. In CpG4.1 and CpG4.2, methylation levels were between 10% and 20%, but no significant differences were observed; whereas, in CpG4.3 methylation levels showed a significant decrease of approximately 5% in somatotropinomas compared to NPs, albeit displaying very high levels in both cases. Similarly, CpG2 showed high methylation levels, although no significant differences were observed between groups.

As a next step, we evaluated the RNA levels of the two genes of interest, *SSTR5* and *SSTR5‐AS1*, in the same cohorts of somatotropinoma and NP samples (Fig. 1C). Interestingly, *SSTR5* was clearly overexpressed in somatotropinoma samples compared to NP tissues, whereas expression levels of *SSTR5‐AS1* gene showed a similar trend but did not exhibit a statistically significant change. Of note, the expression of both genes showed a direct correlation in both NP and somatotropinoma samples (Fig. 1D), which could suggest a possible functional association between these two genes. Conversely, no correlations were observed between the expression of the antisense gene and the SST_5_TMD4 truncated variant of the receptor (Fig. S2).

Expression of *SSTR5* and *SSTR5‐AS1* genes was next compared with methylation levels of the CpG islands overlapping them in the genome. Remarkably, expression of both genes was tightly and inversely correlated with methylation levels of CpG4.3 (Fig. 1E) in somatotropinoma but not in NP samples, whereas they did not show a significant correlation with methylation levels of any of the other CpG islands examined (Fig. S3). CpG4.3 overlaps two functionally relevant regions; the large exon of *SSTR5* wherein noncanonical alternative splicing can take place, and the putative promoter of *SSTR5‐AS1*. Therefore, the methylation at CpG4.3 could be related with the expression of these two genes in somatotropinomas, in a manner that might be relevant to their pathological context. Nonetheless, the methylation levels of this CpG island or any of the others measured in this work did not exhibit correlations with the expression levels of the truncated isoform SST_5_TMD4 (Fig. S4).

In order to investigate whether the relationship between *SSTR5* and its NAT *SSTR5‐AS1* is also present in other tumors where the somatostatin‐SST system is important, we extended our study to PanNETs. To this end, expression of both genes was measured in a cohort of 15 PanNETs, comparing tumor tissue with their paired nontumor adjacent tissue (NTAT), used as reference. Results from this analysis revealed that, while *SSTR5* expression did not differ between both regions, the levels of *SSTR5‐AS1* mRNA were significantly higher in tumor samples (Fig. 1F). By contrast, expression levels of these genes were directly and strongly associated in both tumor and nontumor tissue, reinforcing the idea of a functional link between them (Fig. 1G). Unfortunately, the methylation levels of these samples could not be measured due to the limited quality of the DNA from formalin‐fixed paraffin‐embedded samples.

### 3.2 *SSTR5‐AS1* and *SSTR5* expression levels are interrelated and may be altered by demethylases

**Fig. 2:**
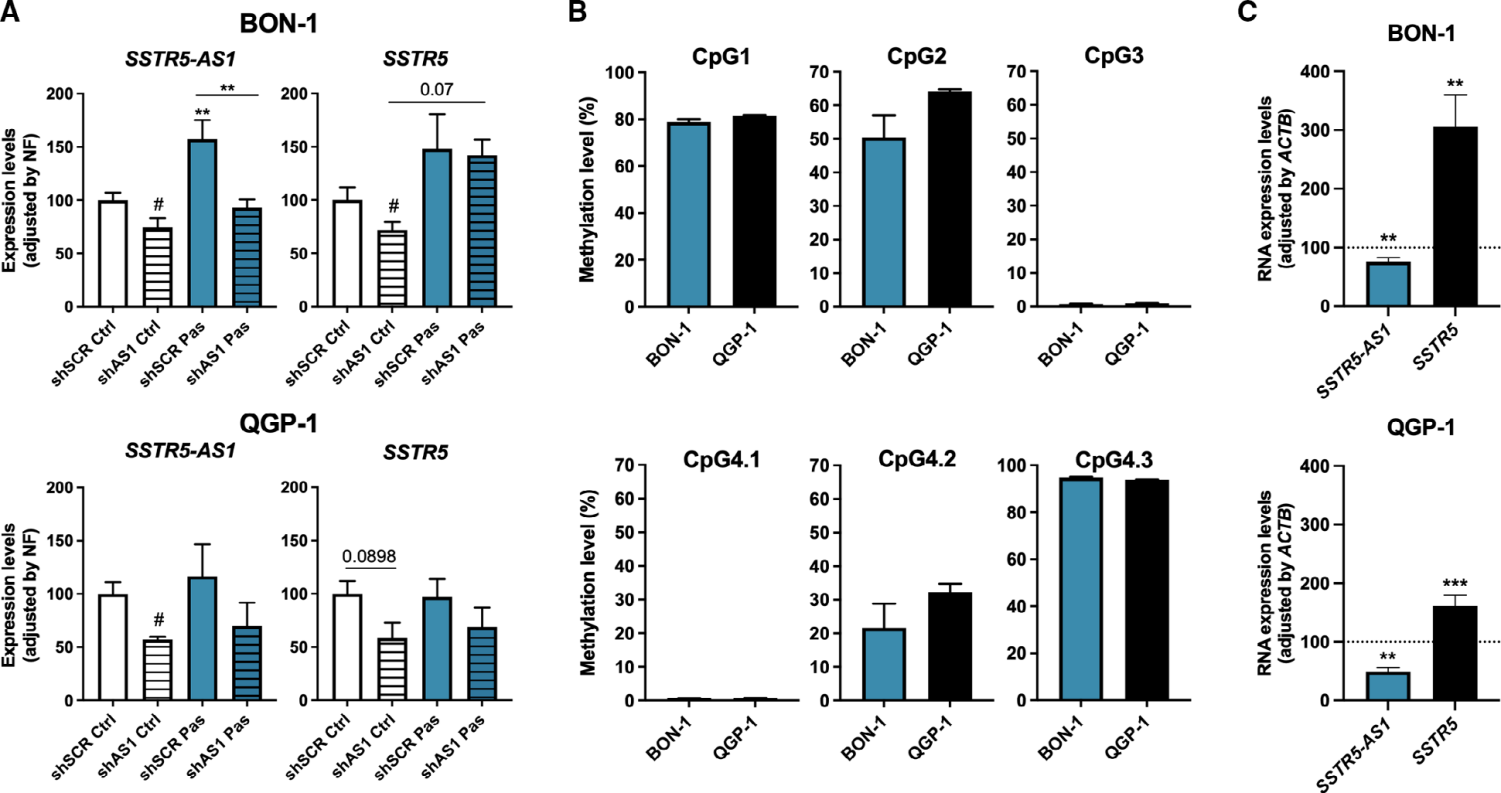
Interrelation of *SSTR5‐AS1* and *SSTR5* expression and regulation by 5‐azacytidine. (A) Expression levels of *SSTR5‐AS1* and *SSTR5* were evaluated in BON‐1 and QGP‐1 cell lines after *SSTR5‐AS1* silencing (striped bars) and 24‐h treatment with pasireotide 100 nm (Pas, blue), and were measured by qPCR, and adjusted by normalization factor (NF) with *ACTB*, *GAPDH* and *HPRT* housekeeping genes. (B) Basal methylation levels of BON‐1 and QGP‐1 in *SSTR5* and *SSTR5‐AS1* loci, expressed as percentage. (C) RNA expression of *SSTR5‐AS1* and *SSTR5* after treatment with 5‐azacytidine demethylase. Asterisks (**P < 0.01; ***P < 0.001) indicate values that significantly differ between groups under one‐way ANOVA; # symbol indicates values that significantly differ from control under *t* test. In all cases, data represent mean ± SEM of *n* = 3 and five independent experiments for BON‐1 and QGP‐1, respectively.

To better understand the potential functional role of *SSTR5‐AS1* in NETs, we performed a stable silencing of this NAT using a specific shRNA and interrogated its possible link with the *SSTR5* gene. For this and the ensuing assays, the PanNET model cell lines BON‐1 and QGP‐1 were used, also due to the lack of suitable human cell models for somatotropinomas. After silencing, cells were treated with pasireotide, a second‐generation SSA with high affinity for SST_5_, in order to test whether *SSTR5‐AS1* may impact in the cell response to this treatment. Interestingly, the first observation was that *SSTR5‐AS1* silencing by 30%, concomitantly decreased *SSTR5* expression in BON‐1 cells (Fig. 2A), and, while not reaching a significant difference, it caused a similar trend to decrease in QGP‐1 cells. The relation between the expression of these two genes seems to be reciprocal, working in both directions, in that silencing of *SSTR5* with a specific siRNA also decreases the expression of *SSTR5‐AS1* (Fig. S5). Treatment with pasireotide (100 nm; 24 h) increased the expression levels of both *SSTR5* and *SSTR5‐AS1* only in BON‐1 cells, suggesting the existence of a positive feedback regulatory mechanism linking SST_5_ activation and the expression of this receptor, which may also involve NAT. Intriguingly, whereas silencing of *SSTR5‐AS1* fully abrogated the stimulatory effect of pasireotide on the expression of this NAT, the same did not occur with *SSTR5*, rather, pasireotide also tended to elevate *SSTR5* expression under NAT silencing.

Next, to further study the possible role of methylation in the expression of *SSTR5* and *SSTR5‐AS1* genes, basal methylation levels of CpG islands of interest were measured in BON‐1 and QGP‐1 cells. Interestingly, both cell lines exhibited similar levels of methylation in all the CpG islands evaluated (Fig. 2B). In fact, their levels were comparable to those observed for the human samples (Fig. 1B), except for CpG1, which displayed higher methylation levels in both cell lines than in human samples; these findings also indicated that this particular zone was more methylated in somatotropinoma samples than in NP. To explore this issue in more detail, cells were treated for 48 h with different doses of the demethylating agent 5‐azacytidine (Fig. S6A). The highest effects were observed with 5 μm 5‐azacytidine, which acted oppositely in both genes, decreasing *SSTR5‐AS1* and increasing *SSTR5* expression levels (Fig. 2C). This finding contrasts with the direct correlation of the expression levels of both genes observed in the previous measurements and may unveil a potential for a distinct epigenetic regulation for each gene. However, despite the ability of 5‐azacytidine treatment to clearly influence gene expression, no specific alterations were found in the methylation of the CpG islands studied (Fig. S6B). These results may suggest that the changes observed are not a direct consequence of a demethylation of *SSTR5*/*SSTR5‐AS1* but may reflect off‐target effects of the dose of 5‐azacytidine used or may be mediated by an indirect influence of trans‐regulatory elements, such as transcription factors. In any case, our findings in the cell lines suggest that DNA methylation may not be a direct regulatory mechanism for the expression of *SSTR5*/*SSTR5‐AS1* but may influence it indirectly.

### 3.3 Decrease in *SSTR5‐AS1* expression promotes aggressiveness features *in vitro*

**Fig. 3:**
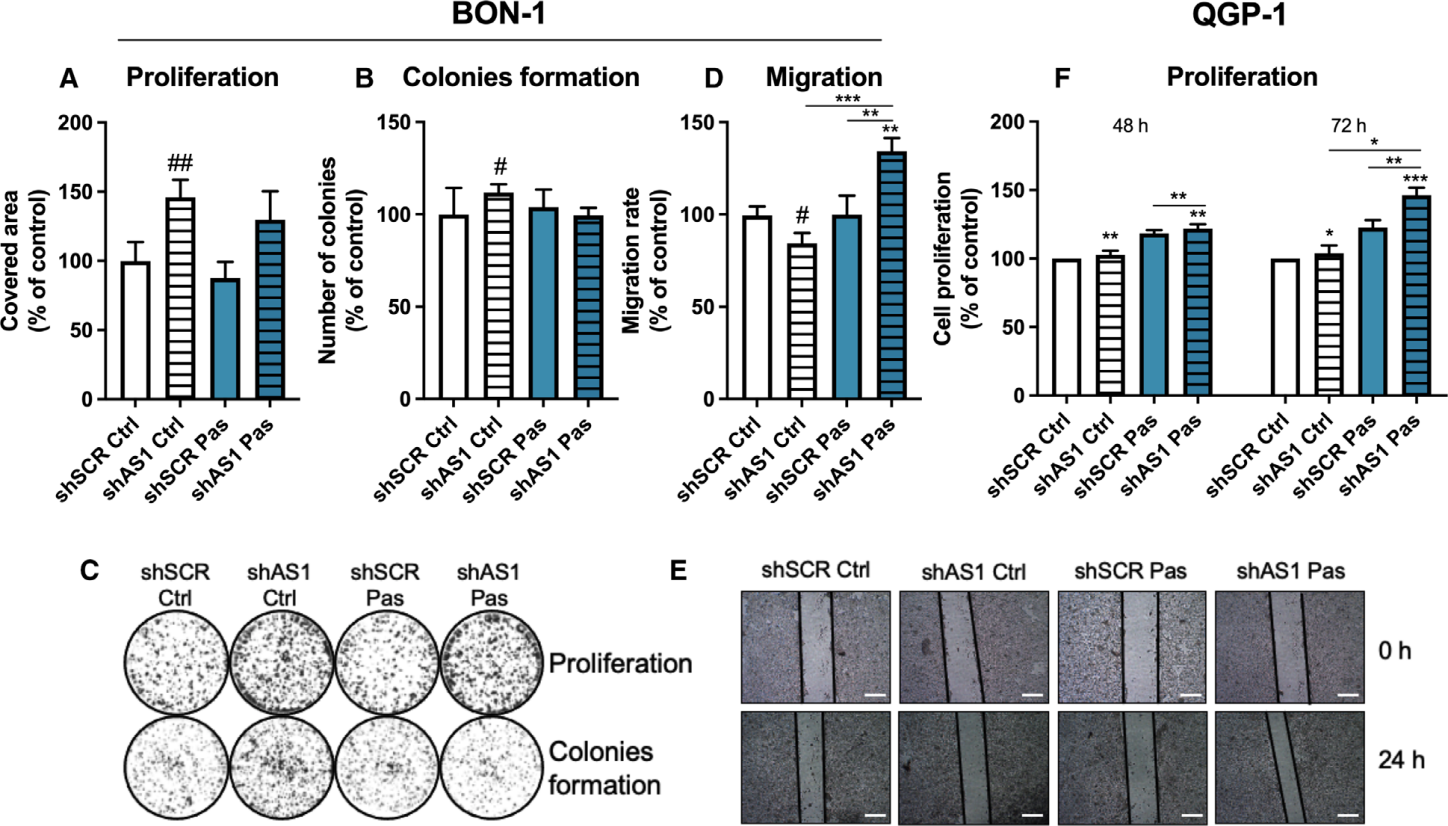
Alteration of aggressiveness features after *SSTR5‐AS1* silencing in BON‐1 and QGP‐1. (A) Proliferative rate of BON‐1 cells after 10 days of silencing (striped bars) and/or pasireotide treatment (Pas, blue), represented as area covered in the well. (B) Capacity to form colonies under *SSTR5‐AS1* silencing (striped bars) and/or 24 h of pretreatment with pasireotide (blue), measured by number of colonies after 10 days of incubation. (C) Representative pictures of proliferation (top) and colonies formation (bottom) assays in BON‐1 cells. (D) Migration rate under *SSTR5‐AS1* silencing (striped bars) and/or pasireotide treatment (blue), after 24 h of the wound, represented by healed area. (E) Representative pictures of migration assay in BON‐1, scale bars represent 500 μm. (F) Proliferative rate of QGP‐1 cells after 48 and 72 h. Asterisks (*P < 0.05; **P < 0.01; ***P < 0.001) indicate values that significantly differ between groups under one‐way ANOVA; # symbols indicate values that significantly differ from control under *t* test. In all cases, data are presented as percentage of control and represent mean ± SEM of *n* = 5 independent experiments for proliferation and 6 for colonies formation and migration.

To further examine the functional role of *SSTR5‐AS1*, we tested whether the presence of this NAT influences tumor aggressiveness features *in vitro* using the BON‐1 and QGP‐1 cell models. Specifically, proliferation was measured in these cell lines, while colony formation and migration were measured in BON‐1, under *SSTR5‐AS1* silencing and pasireotide treatment. This approach first showed that NAT silencing clearly increased cell proliferation under basal culture conditions. Conversely, pasireotide did not alter proliferation under basal conditions, while it seemingly blunted the effect of NAT silencing (Fig. 3A). Interestingly, colony formation was also elevated after *SSTR5‐AS1* silencing, as compared to its scramble control, further suggesting the ability of this NAT to influence malignancy features of NET cells. Conversely, pasireotide did not alter colony formation under control conditions, while, again, blunting the stimulatory action of NAT silencing (Fig. 3B). In contrast with the above, *SSTR5‐AS1* silencing did not increase but decreased cell migration, compared to scramble shRNA, thereby suggesting a disconnection between the actions of *SSTR5‐AS1* on these distinct functional cell features. Of note, pasireotide, while, as in the previous parameters measured, did not alter migration under control conditions (scramble shRNA), surprisingly increased migration when *SSTR5‐AS1* was silenced (Fig. 3D). We were also able to evaluate cell proliferation on QGP‐1 cells, and we observed that NAT silencing also increased cell proliferation under basal conditions (Fig. 3F); moreover, after NAT silencing, pasireotide exerted an additional stimulatory effect in this cell line, which is reminiscent of the results found in migration studies on BON‐1 cells. These observations highlight the relevance of the consequences that changes in *SSTR5‐AS1* expression may impact on the function of *SSTR5* gene; in fact, proliferation assays performed after *SSTR5* silencing resulted in similar, consistent increases in both cell lines (Fig. S7).

**Fig. 4:**
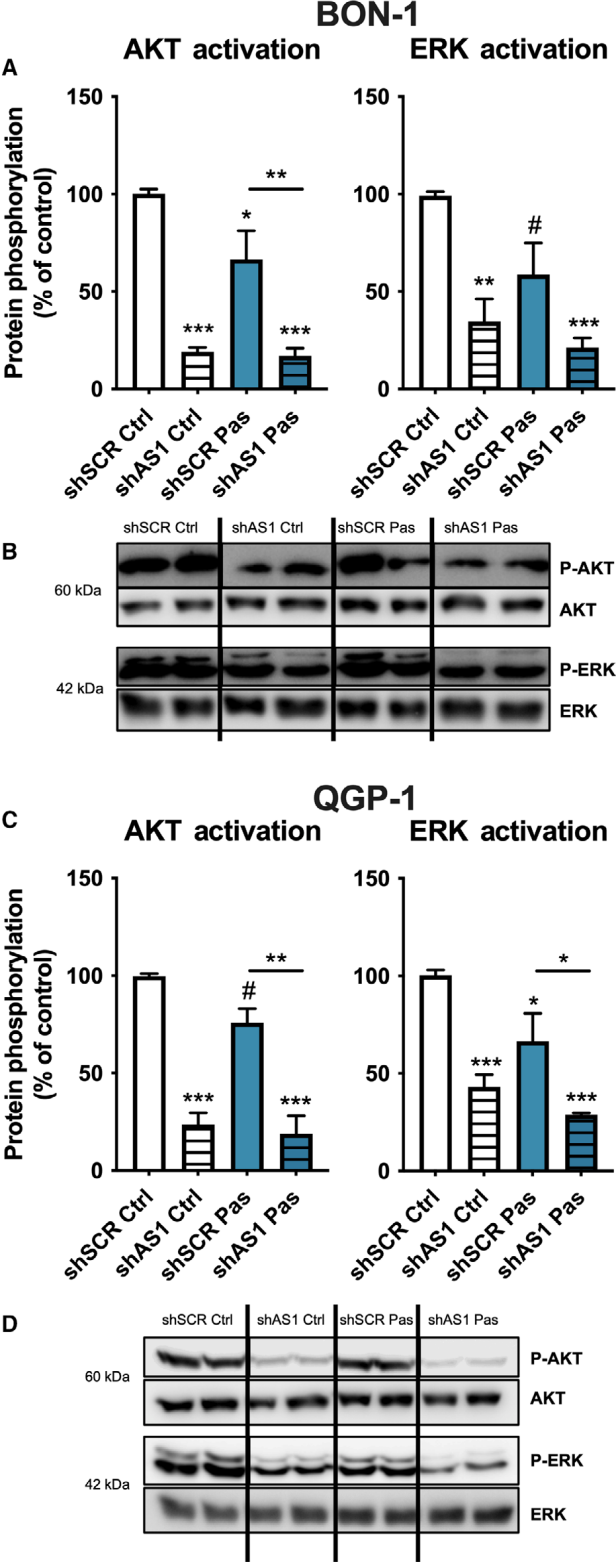
Silencing of *SSTR5‐AS1* alters key SST_5_‐related signaling pathways in BON‐1 (A, B) and QGP‐1 (C, D) cells. Protein phosphorylation of AKT and ERK in both cell lines after *SSTR5‐AS1* silencing (striped bars) and after 10 min of pasireotide treatment (Pas, blue). This activation was measured by western blot and normalized with total AKT/ERK. Asterisks (*P < 0.05; **P < 0.01; ***P < 0.01) indicate values that significantly differ between groups (one‐way ANOVA analysis); # symbol indicates values that significantly differ from control under *t* test. In all cases, data represent mean ± SEM of *n* = 4 independent experiments.

In line with this, we finally evaluated the impact of *SSTR5‐AS1* on the activation of key proteins within typical signaling pathways regulated by SST_5_. Thus, activation of AKT and ERK were assessed after *SSTR5‐AS1* silencing and after 10 min of pasireotide treatment. Results obtained showed that NAT silencing decreased both AKT and ERK activation, compared to scramble shRNA (Fig. 4). Interestingly, pasireotide treatment exerted a slight but significant effect decreasing both AKT and ERK phosphorylation in BON‐1 and QGP‐1 under control conditions (scramble shRNA). Furthermore, pasireotide was unable to appreciably modify their phosphorylation levels after *SSTR5‐AS1* silencing.

## 4 Discussion

There is now ample evidence that the somatostatin system plays a key pathophysiological role in various tumors, particularly in NETs, where detection of specific SSTs and use of synthetic SSAs provide valuable diagnostic and therapeutic tools [16]. SSAs are currently used to control tumor growth and/or hormone secretion in somatotropinomas (and other PitNETs) and in PanNETs, when surgery is not amenable [17, 28, 50]. SSAs action in these tumors requires sufficient SSTs expression, particularly SST_2_, the primary target of first‐generation SSAs, lanreotide and octreotide [16]. Unfortunately, an appreciable proportion of patients are unresponsive to SSAs or develop resistance [16, 29]. However, although it might apparently represent a survival disadvantage for the tumor, NETs also express high levels of other SSTs, especially SST_5_, which would enable the use of alternative treatments. Indeed, although first‐generation SSAs bind SST_5_ with high affinity, this receptor is a better target for the second‐generation SSA pasireotide [51]. In fact, this SSA is currently used for the treatment of certain patients in different types of NETs [52, 53].

The biology of SST_5_ differs substantially from that of SST_2_ or the other SSTs, and is still far from being fully understood [16, 54]. High SST_5_/SST_2_ ratio has been linked to SSAs resistance in acromegaly [33, 55, 56]. Likewise, human *SSTR5* is the only gene of the SSTR family that, despite lacking typical introns in its coding sequence, can generate aberrant splice variants, for example, SST_5_TMD4, which are overexpressed in NETs and have been linked to oncogenic processes and SSAs resistance [16, 25, 33]. This underscores the importance of advancing in our understanding of the mechanisms regulating *SSTR5* expression and the biogenesis of SST_5_, and to identify putative factors controlling its functioning in NETs.

In this scenario, we initially applied an *in silico* analysis of the *SSTR5* gene region that revealed the existence of a natural antisense transcript (NAT) overlapping in the genome with *SSTR5* gene, which had already been named, accordingly, *SSTR5‐AS1*, but whose role or regulation had not yet been reported. A closer analysis revealed that, distributed along the loci of these two genes, there are four CpG islands which could be targets for DNA methylation. We then analyzed in detail these two original features of *SSTR5* in NETs. Specifically, presence and relative abundance of *SSTR5‐AS1* with respect to *SSTR5* was examined in somatotropinomas and PanNETs, whereas methylation levels of the different islands were measured in two PanNET cell lines and in the cohort of somatotropinomas. Results from this latter approach revealed, for the first time, that some of these CpG islands were differentially methylated in somatotropinomas, compared with normal pituitary (NP). Specifically, the CpG island overlapping the last exon of the NAT gene *SSTR5‐AS1* was more methylated in somatotropinomas than in NP, whereas the one overlapping the first exon of *SSTR5,* and its putative promoter was hypomethylated in somatotropinomas compared to NP. The most distant part, overlapping the area where alternative splicing is presumed to occur, in the middle of the large exon of *SSTR5* and the putative NAT promoter, was significantly less methylated in somatotropinomas than in NP. Moreover, methylation levels of CpG4.3 were tightly associated with *SSTR5* and *SSTR5‐AS1* expression in somatotropinomas, where lower levels of methylation were linked to higher expression of these genes, but not in NP samples. These findings suggest that methylation of this CpG island could be related to the expression of these two genes in a pathologically relevant context, which is in line with results from a recent study that examined *SSTR5*/*SSTR5‐AS1* in laryngeal carcinoma [38]. However, although the treatment with the demethylating agent 5‐azacitydine clearly altered the expression of both genes in the cell lines studied, no specific changes were observed in the methylation of the specific CpG islands analyzed; therefore, further studies are warranted to test whether these observations also occur in primary tumors and to precisely dissect the mechanisms underlying the observed changes, which might derive from off‐target and/or indirect effects of the demethylating agent, and, in turn, would suggest that methylation may not be directly, but indirectly involved in *SSTR5*/*SSTR5‐AS1* expression. In particular, the lack of association between methylation in CpG4.3 and *SSTR5*/*SSTR5‐AS1* expression in NP is intriguing and could suggest a differential regulatory role of this interaction in normal somatotropes, or a distinct contribution of the heterogeneous cell population comprising healthy pituitary tissue, compared to the monoclonal tumor somatotrope population comprising GH‐secreting tumors. Nonetheless, the present findings provide novel cues to further explore and understand the regulation of *SSTR5* expression in tumor somatotropes and other tumor and normal cell types.

There is increasing interest in NATs given their ability to regulate the expression of their sense genes [36]. Consequently, we analyzed the expression of *SSTR5‐AS1* and its relationship with that of *SSTR5* on the same cohort of somatotropinoma samples as well as in an additional set of PanNETs. Interestingly, *SSTR5‐AS1* expression in PanNETs was higher in tumor tissue as compared to the nontumor adjacent tissue. In contrast, no such differences were found in somatotropinomas compared to NP. However, in both PitNETs and PanNETs, as well as in their respective control tissues, we discovered an interesting common behavior: there was a tight, direct association between the expression of *SSTR5‐AS1* and that of *SSTR5*. These results are in agreement with the findings reported in laryngeal carcinoma [38], and support a close relationship between the control of both genes, which may involve a regulation by common factors, but also a direct interaction of the two genes during their expression. This latter mechanism is likely to be in place, in that our results not only proved that silencing of *SSTR5‐AS1* caused a marked decrease in *SSTR5* expression *in vitro,* but also silencing of *SSTR5* caused a decrease in *SSTR5‐AS1* in BON‐1 and QGP‐1 cells.

We next sought to further understand the precise functional role of *SSTR5‐AS1* gene in NETs, by evaluating different mechanistic endpoints on the PanNET BON‐1 and QGP‐1 cell models after silencing this NAT. This approach revealed that *SSTR5‐AS1* silencing had a profound functional impact, as it increased cell proliferation and/or colony formation in BON‐1 and QGP‐1 cells. This fact may appear somewhat counterintuitive, since this gene is overexpressed in tumoral tissues; however this observation is likely linked to the inhibition of *SSTR5* expression mentioned above, since this receptor can exert antitumor functions and has been shown to have ligand‐independent constitutive activity, as it is suggested by the results of the proliferation assay after silencing *SSTR5* and as it has been reported in the literature [16, 18, 57]. In contrast, *SSTR5‐AS1* silencing caused a decrease in cell migration, apparently implying that this NAT, either directly or through SST_5_ could contribute to sustain the migratory capacity of BON‐1 cells under basal culture conditions. These observations unveil an apparent divergence between two typical tumor features, in that a reduction in the expression of this NAT would concomitantly increase proliferation but decrease migration. Obviously, it would be of interest to explore whether these actions caused by the partial loss of *SSTR5‐AS1* bear similar consequences *in vivo*, particularly in tumors. These seemingly opposing actions may involve a distinct ability of *SSTR5‐AS1* to influence downstream signaling, as its silencing decreased activation of AKT and ERK, two key players in pathways controlling multiple cell functions and with a complex cross‐talking regulatory network. Typically, AKT and ERK inhibition are related with antitumor actions [58, 59], which would be in keeping with the downregulation of migration observed after *SSTR5‐AS1* silencing. In fact, these pathways have been previously related with *SSTR5* in the literature [16]. However, these reductions would not similarly fit with the increased proliferation and colony forming assays, thus suggesting that additional mechanisms must be in place underlying these actions and, therefore, that further studies are necessary to fully elucidate the mechanisms mediating *SSTR5‐AS1* function.

A final set of studies was aimed to ascertain whether *SSTR5‐AS1* may influence the response of BON‐1 and QGP‐1 cells to the SST_5_‐preferring SSA pasireotide. Interestingly, pasireotide treatment increased *SSTR5* expression in BON‐1 cells, similar to that previously reported by our group in pituitary tumor cells [43]. But, most importantly, pasireotide also increased *SSTR5‐AS1* expression, which could imply that the positive feedback between SST_5_ activation and expression of this receptor may involve or, at least be related to, that of the NAT itself. This effect was not observed in QGP‐1 cells, probably due to the different origin of these two cell lines, as underscored by recent studies indicating that these cells are molecularly and functionally different [39]. In fact, presence of *SSTR5‐AS1* shRNA impaired pasireotide to increase NAT expression in BON‐1 cells but not in QGP‐1 cells; this differential action was not only cell type‐dependent but also gene‐dependent, as NAT silencing did not seem to fully abrogate the ability of pasireotide to upregulate *SSTR5* expression in BON‐1 cells. Moreover, in keeping with our previous findings in PanNET cell lines [22, 60, 61], the functional and signaling actions of pasireotide in these cells were limited in terms of cell proliferation and protein activation, as it did not alter most of the parameters measured, nor was able to overcome the reduction in AKT and ERK activation caused by *SSTR5‐AS1* silencing. Oddly enough, under this silencing pasireotide stimulated cell migration in BON‐1 cells, while it had no effect in nonsilenced control cells. These results are different from those reported on other NET cells expressing *SSTR5*, as it is the case of PitNET cells reported by Peverelli *et al*. [62], where pasireotide significantly decreased cell migration of GH3 cell line and human primary PitNET cell cultures. These apparent discrepancies may be related to the marked biological differences between PitNET and PanNET, in that in BON‐1 cells, a typical model from the latter, derived from aggressive cells from a lymph node metastasis of a NET, we observed that pasireotide did not have any effect on ERK or AKT activation. These results, together with the increased proliferation in response to pasireotide in QGP‐1 cells, confirm the unexpected but limited ability of pasireotide to influence key functional parameters in PanNETs bearing SST_5_ and, at the same time, unveil an association between SST_5_ activation, expression of *SSTR5* and its NAT, *SSTR5‐AS1*, and the actions of pasireotide on key features in cancer cells, proliferation, and migration, which warrant further investigations in PanNETs cells.

## 5 Conclusions

In summary, our study uncovers two novel mechanisms that may be related to the regulation of *SSTR5* expression in cells from PanNETs and somatotropinomas, namely, differential methylation of intragenic regions and post‐transcriptional events mediated by *SSTR5‐AS1*. The results presented herein reveal that methylation of specific *SSTR5* gene CpG regions may be, at least indirectly, associated to the upregulation of both *SSTR5* and *SSTR5‐AS1* expression. While *SSTR5‐AS1* clearly influences *SSTR5* and *SSTR5‐AS1* expression as well as promotes NET cell aggressiveness features, including proliferation, migration, and colony formation, and can be involved in the limited response of PanNET cells to pasireotide. However, the precise contribution of these new regulatory mechanisms of SST_5_ biology to the clinical behavior and pharmacological response of pituitary and pancreatic NETs as well as other tumors warrants and awaits future elucidation.

## Conflict of interest

The authors declare no conflict of interest.

## Author contributions

SPA, AIC, MC, RML, and JPC conceptualized the project; SPA, AIC, RBE, MCVB, MRB, and MC involved in experiment performance; SPA, AIC, RBE, MCVB, MRB, MDG, MC, RML, and JPC analyzed the data; ADHM, EVM, MAGM, and ASM involved in samples acquisition; SPA, AIC, MC, RML, and JPC wrote manuscript preparation; SPA, AIC, RBE, MRB, ADHM, EVM, MAGM, MK, ASM, MDG, MC, RML, and JPC reviewed and edited the document; MC, RML, and JPC contributed to funding acquisition.

### Peer Review

The peer review history for this article is available at https://publons.com/publon/10.1002/1878‐0261.13107.

## Supporting information


**Fig. S1.** UCSC Genome Browser (version GRCh37/hg19) representation of *SSTR5‐AS1* and *SSTR5* loci.

**Fig. S2.** Correlations of *SSTR5‐AS1* and *SST_5_TMD4* expression in NP and somatotropinoma samples, measured by qPCR and normalized by *ACTB*.

**Fig. S3.** Correlations between methylation levels of CpGs and expression levels of *SSTR5* and *SSTR5‐AS1* in NP and somatotropinoma samples.

**Fig. S4.** Correlations between methylation levels of CpGs and expression levels of *SST_5_TMD4* in NP and somatotropinoma samples.

**Fig. S5.** RNA expression of *SSTR5* and *SSTR5‐AS1* after *SSTR5* silencing compared to scramble siRNA (100%).

**Fig. S6.** A. RNA expression of *SSTR5* and *SSTR5‐AS1* after treatment with different doses of 5‐azacytidine in BON‐1 and QGP‐1. B. Methylation levels of CpGs in cell lines treated with 5‐azacytidine, compared to nontreated control. Asterisks (*, p < 0.05; **, p < 0.01; ***, p < 0.001) indicate values that significantly differ from control under ANOVA analysis.

**Fig. S7.** Proliferation assay after *SSTR5* silencing in BON‐1 and QGP‐1 cell lines, performed with Alamar Blue. Asterisks (*, p < 0.05; **, p < 0.01) indicate values that significantly differ from control under *t* test. Data are presented as percentage of control.

**Table S1.** Summary of clinical parameters of somatotropinoma and PanNETs patients.
**Table S2.** Details of primers used for quantitative PCR (a), as well as methylation assays (b).

## Data Availability

The data that support the findings of this study are available from the corresponding authors [justo@uco.es; raul.luque@uco.es; b12ibcoa@uco.es] upon reasonable request.
