# Supplementary figures and images for: Epigenetic and post‐transcriptional regulation of somatostatin receptor subtype 5 (SST5) in pituitary and pancreatic neuroendocrine tumors
Source: Mol Oncol. 2021 Oct 26;16(3):764–79. doi: 10.1002/1878-0261.13107 (PMC8807362; doi:10.1002/1878-0261.13107)

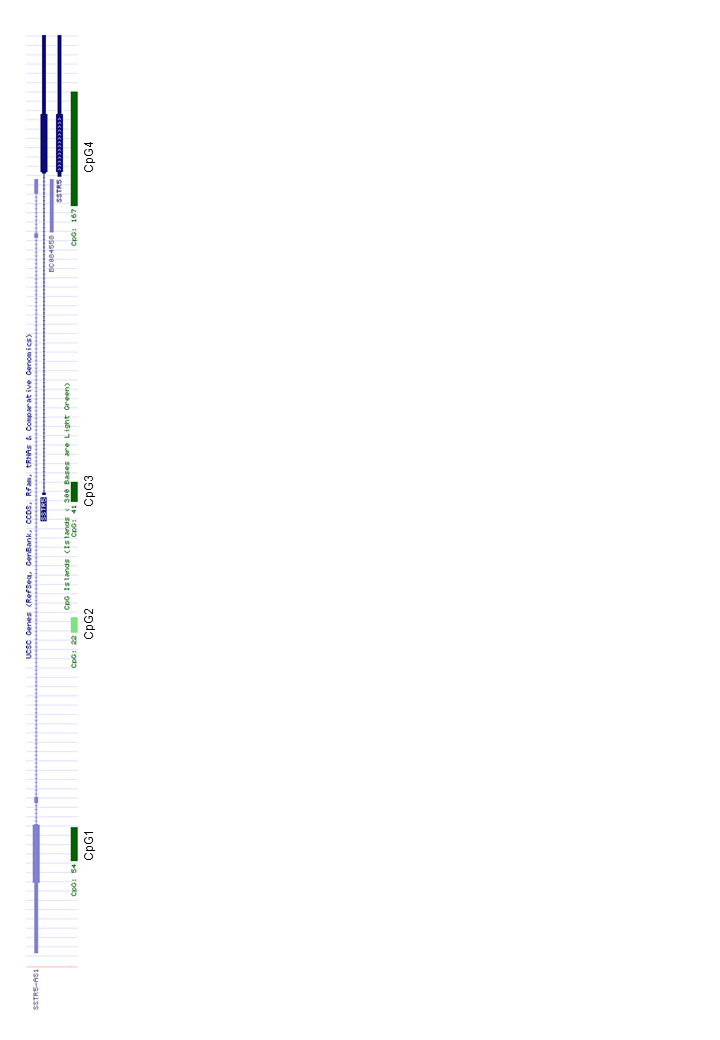

Supplement: Supplementary file 1 — Fig. S1. UCSC Genome Browser (version GRCh37/hg19) representation of SSTR5‐AS1 and SSTR5 loci. [file MOL2-16-764-s003.tif]

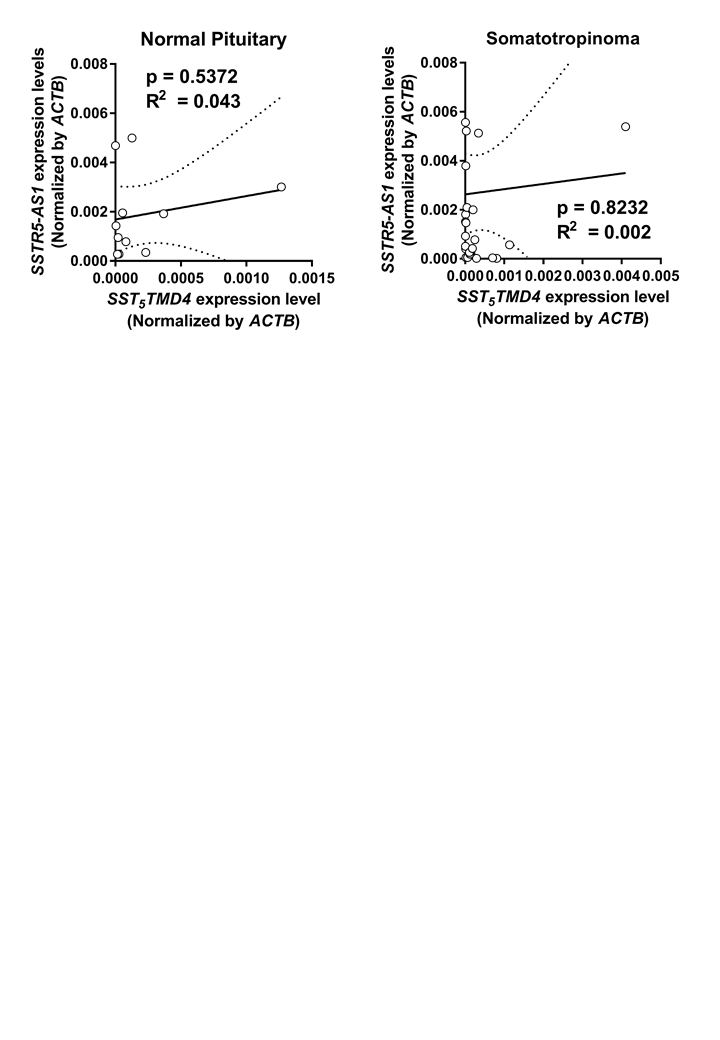

Supplement: Supplementary file 2 — Fig. S2. Correlations of SSTR5‐AS1 and SST5TMD4 expression in NP and somatotropinoma samples, measured by qPCR and normalized by ACTB. [file MOL2-16-764-s001.TIF]

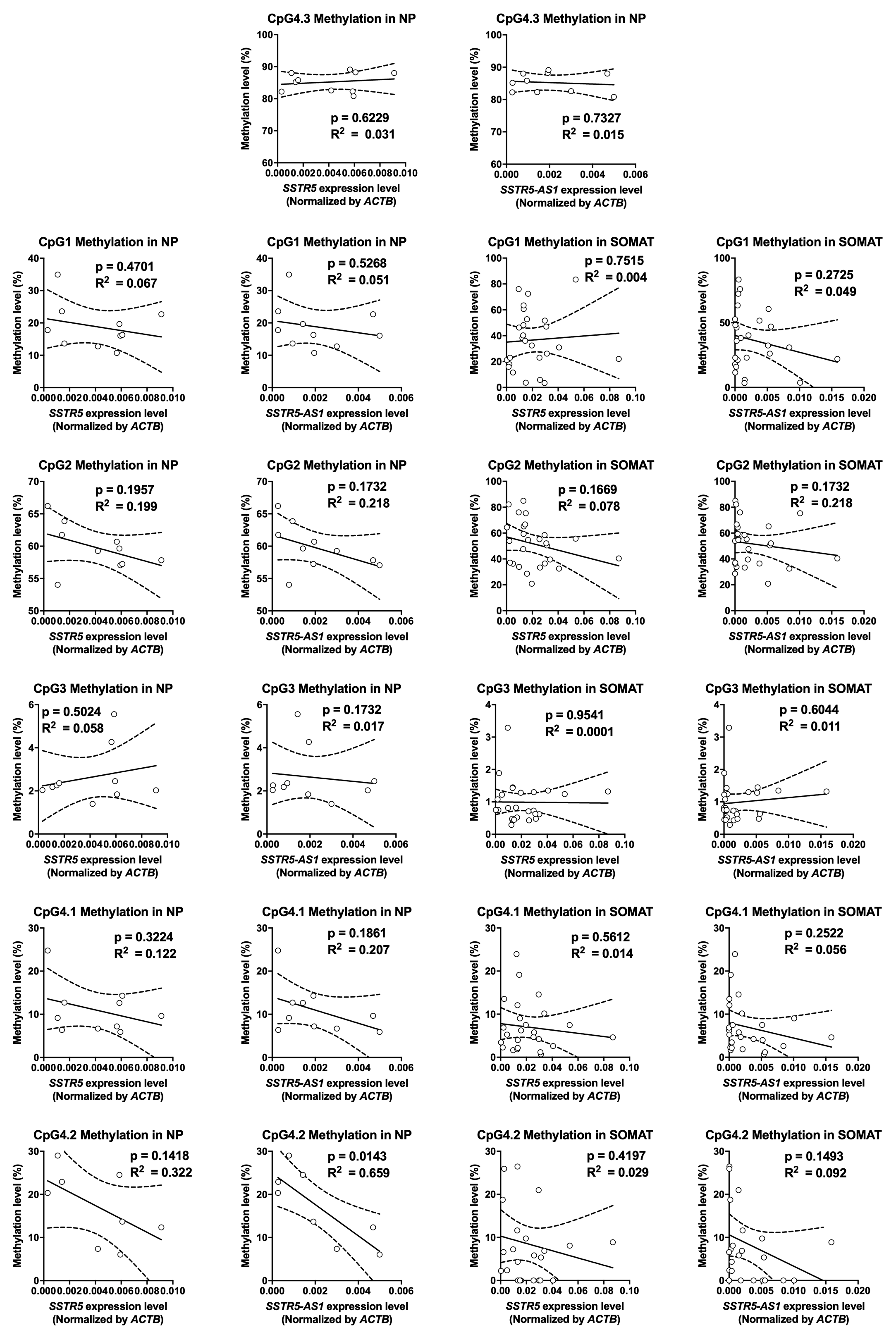

Supplement: Supplementary file 3 — Fig. S3. Correlations between methylation levels of CpGs and expression levels of SSTR5 and SSTR5‐AS1 in NP and somatotropinoma samples. [file MOL2-16-764-s004.tiff]

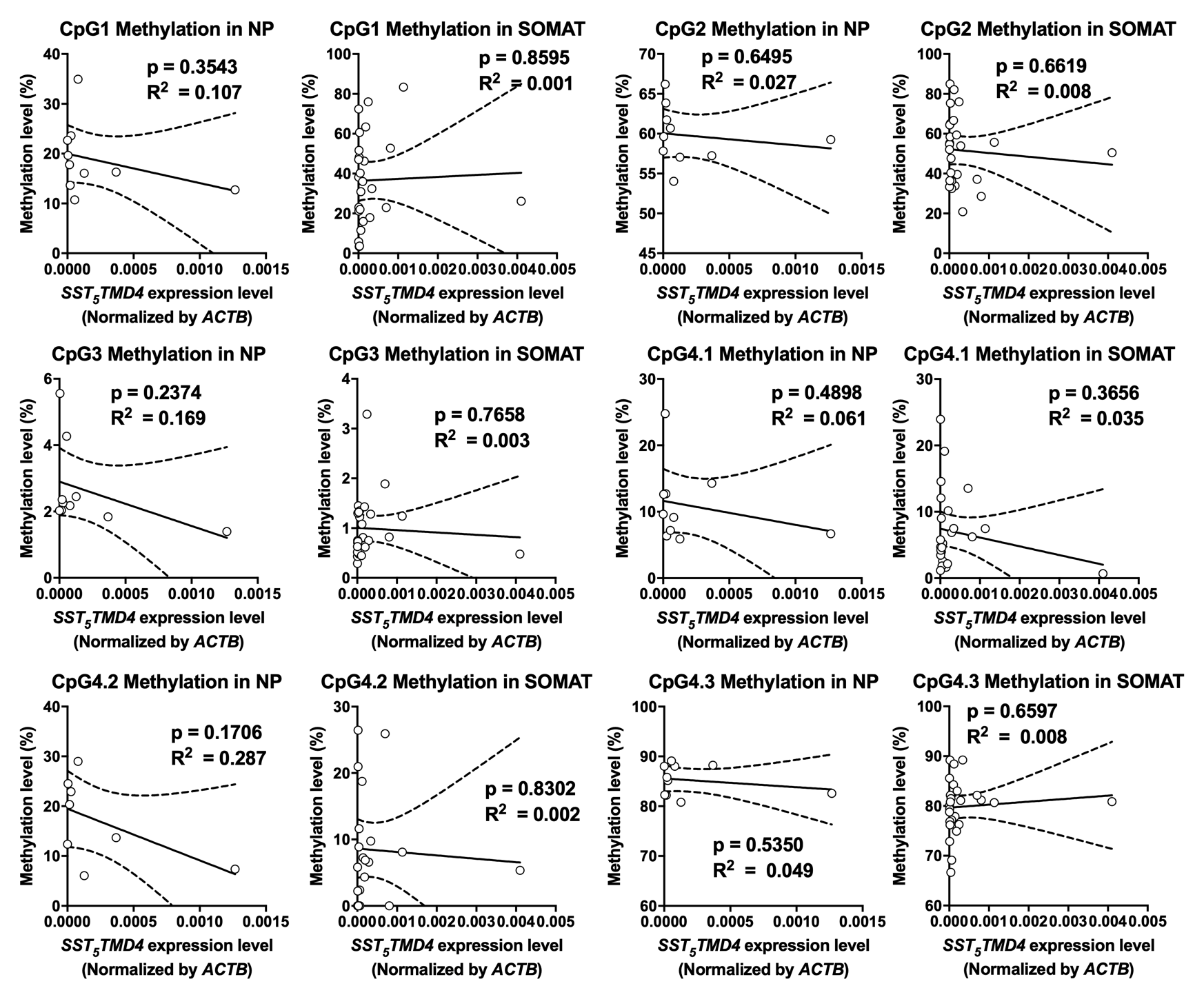

Supplement: Supplementary file 4 — Fig. S4. Correlations between methylation levels of CpGs and expression levels of SST5TMD4 in NP and somatotropinoma samples. [file MOL2-16-764-s006.tiff]

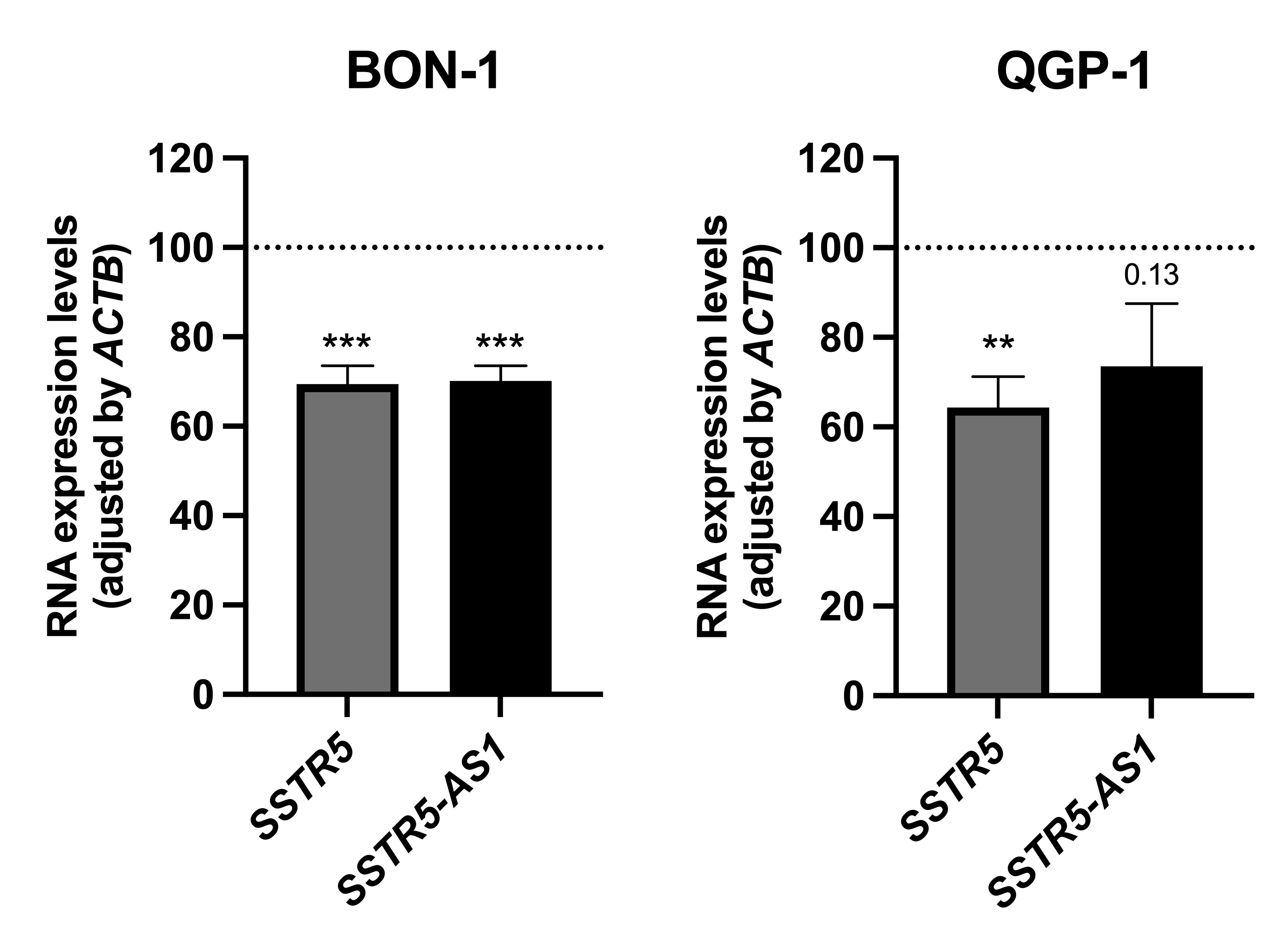

Supplement: Supplementary file 5 — Fig. S5. RNA expression of SSTR5 and SSTR5‐AS1 after SSTR5 silencing compared to scramble siRNA (100%). [file MOL2-16-764-s008.tiff]

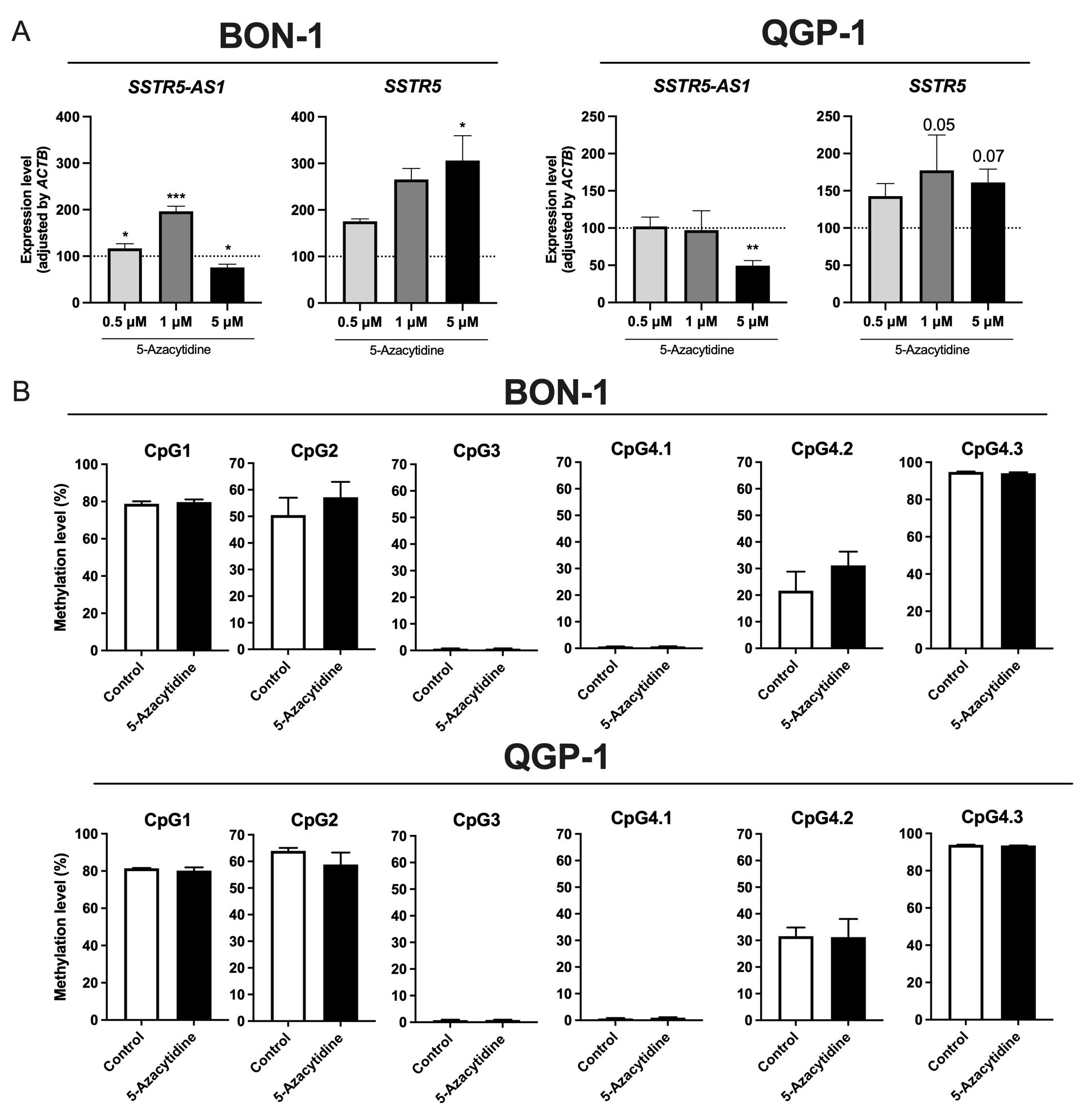

Supplement: Supplementary file 6 — Fig. S6. A. RNA expression of SSTR5 and SSTR5‐AS1 after treatment with different doses of 5‐azacytidine in BON‐1 and QGP‐1. B. Methylation levels of CpGs in cell lines treated with 5‐azacytidine, compared to nontreated control. Asterisks (*, p < 0.05; **, p < 0.01; ***, p < 0.001) indicate values that significantly differ from control under ANOVA analysis. [file MOL2-16-764-s002.tiff]

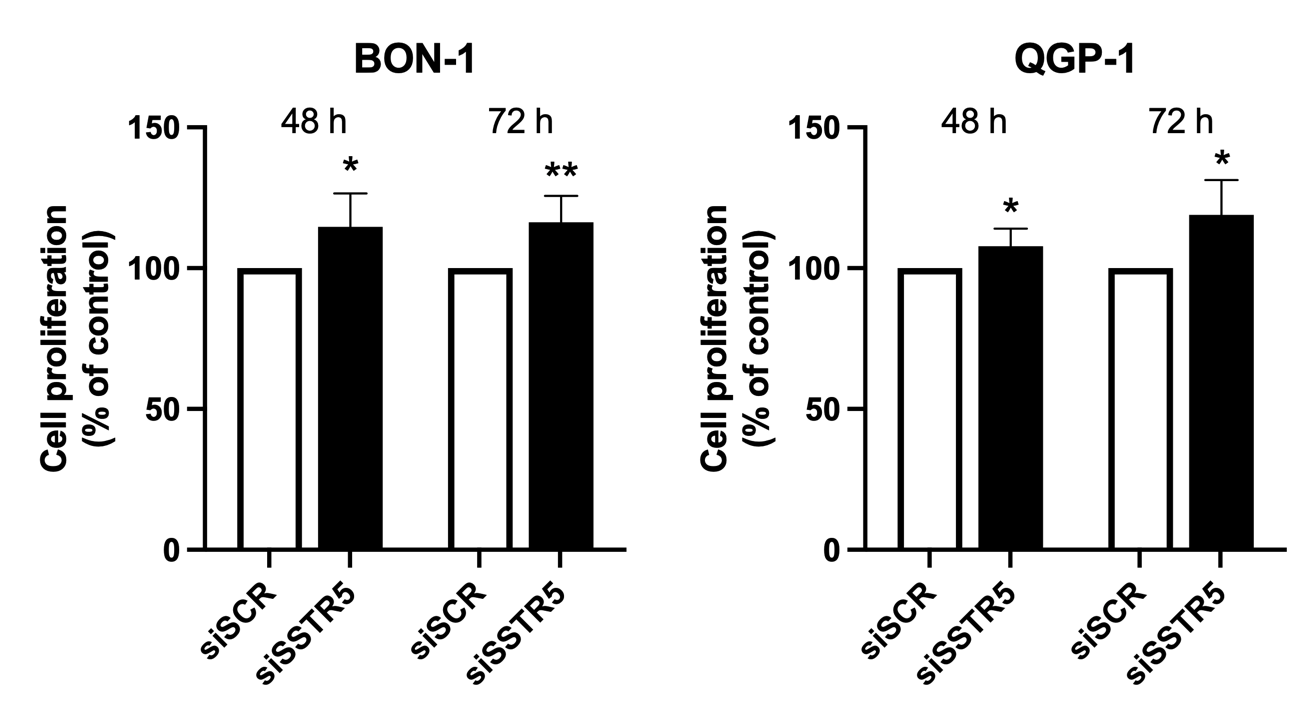

Supplement: Supplementary file 7 — Fig. S7. Proliferation assay after SSTR5 silencing in BON‐1 and QGP‐1 cell lines, performed with Alamar Blue. Asterisks (*, p < 0.05; **, p < 0.01) indicate values that significantly differ from control under t test. Data are presented as percentage of control. [file MOL2-16-764-s005.tiff]
