# Supplementary material for: Epigenetic and post‐transcriptional regulation of somatostatin receptor subtype 5 (SST5) in pituitary and pancreatic neuroendocrine tumors
Source: Mol Oncol. 2021 Oct 26;16(3):764–79. doi: 10.1002/1878-0261.13107 (PMC8807362; doi:10.1002/1878-0261.13107)
Supplement: Supplementary file 8 — Table S1. Summary of clinical parameters of somatotropinoma and PanNETs patients. Table S2. Details of primers used for quantitative PCR (a), as well as methylation assays (b). [file MOL2-16-764-s007.docx]

| **Parameter** | **Somatotropinomas** | **PanNETs** |
| --- | --- | --- |
| **Number of samples** | 27 | 15 |
| **Age (years, mean ± SEM)** | 43 ± 11 | 55 ± 10 |
| **Body Mass Index (kg/m^2^, mean ± SEM)** | 31.0 ± 6.8 | 30.0 ± 3.3 |
| **Gender (female, %)** | 63.2 | 53.3 |
| **IGF-1 (ng/ml, median ± SD)** | 741,7 ± 245,2 | - |
| **Grade (G1/G2, %)** | - | 53.3/46.7 % |

**Supplemental table 1.** Summary of clinical parameters of somatotropinoma and PanNETs patients.

| **Supplemental table 2a** | | | | |
| --- | --- | --- | --- | --- |
| **Transcript** | **Primers application** | **Forward** | **Reverse** | **Size (bp)** |
| *RNA18S1* | qPCR | CCCATTCGAACGTCTGCCCTATC | TGCTGCCTTCCTTGGATGTGGTA | 136 |
| *ACTB* | qPCR | ACTCTTCCAGCCTTCCTTCCT | CAGTGATCTCCTTCTGCATCCT | 176 |
| *GAPDH* | qPCR | AATCCCATCACCATCTTCCA | AAATGAGCCCCAGCCTTC | 122 |
| *HPRT* | qPCR | CTGAGGATTTGGAAAGGGTGT | TAATCCAGCAGGTCAGCAAAG | 157 |
| *SSTR5* | qPCR | CTGGTGTTTGCGGGATGTT | GAAGCTCTGGCGGAAGTTGT | 183 |
| *SSTR5-AS1* | qPCR | AGCACAGGTGTTTCTGCTTCT | CCCTGCTCTGTCTTTCTCGT | 116 |

| **Supplemental table 2b** | |
| --- | --- |
| **CpG** | **DNA METHYLATION PRIMERS** |
| CpG1 Fw | ACACTCTTTCCCTACACGACGCTCTTCCGATCTGGTTGGGGGATGAAGAGT |
| CPG1 Rv | TGACTGGAGTTCAGACGTGTGCTCTTCCGATCTTAAACTCCCCAAACCCAACAAATAAA |
| CPG2 Fw | ACACTCTTTCCCTACACGACGCTCTTCCGATCTAGGATGTTAGGGTATTTTGTGTTTT |
| CPG2 Rv | TGACTGGAGTTCAGACGTGTGCTCTTCCGATCTCCCCCAACAACCTACAAATATTC |
| CPG3 Fw | ACACTCTTTCCCTACACGACGCTCTTCCGATCTGGGTTATTGTTAGTGGGATTAGG |
| CPG3 Rv | TGACTGGAGTTCAGACGTGTGCTCTTCCGATCTACACAAAATAAAACCCCCAATAAAAAT |
| CPG4.1 Fw | ACACTCTTTCCCTACACGACGCTCTTCCGATCTGGTAGTAGTATTGTAGGGTAGGT |
| CPG4.1 Rv | TGACTGGAGTTCAGACGTGTGCTCTTCCGATCTACATACAAACATTCCTTCCTCCTAAA |
| CPG4.2 Fw | ACACTCTTTCCCTACACGACGCTCTTCCGATCTTGTATGTGTTGGTTTAGGGATTTATTA |
| CPG4.2 Rv | TGACTGGAGTTCAGACGTGTGCTCTTCCGATCTACCAAAAAAAACAACCCCAACATAT |
| CPG4.3 Fw | ACACTCTTTCCCTACACGACGCTCTTCCGATCTAGAGTTTTTAGAAGGTTTTGTGTTTT |
| CPG4.3 Rv | TGACTGGAGTTCAGACGTGTGCTCTTCCGATCTCCTAACTTCAACCAACTCTATCC |

**Supplemental table 2**. Details of primers used for quantitative PCR (**a**), as well as methylation assays (**b**).
